# Cathepsin S Deficiency Results in Abnormal Accumulation of Autophagosomes in Macrophages and Enhances Ang II–Induced Cardiac Inflammation

**DOI:** 10.1371/journal.pone.0035315

**Published:** 2012-04-30

**Authors:** Lili Pan, Yulin Li, Lixin Jia, Yanwen Qin, Guanming Qi, Jizhong Cheng, Yongfen Qi, Huihua Li, Jie Du

**Affiliations:** 1 Beijing An Zhen Hospital Affiliated to Capital Medical University, Beijing Institute of Heart, Lung, and Blood Vessel Diseases, Beijing, China; 2 The Key Laboratory of Remodeling-Related Cardiovascular Diseases, Ministry of Education, Beijing, China; 3 Department of Pathology, Capital Medical University, Beijing, China; University Medical Center Utrecht, The Netherlands

## Abstract

**Background:**

Cathepsin S (Cat S) is overexpressed in human atherosclerotic and aneurysmal tissues and may contributes to degradation of extracellular matrix, especially elastin, in inflammatory diseases. We aimed to define the role of Cat S in cardiac inflammation and fibrosis induced by angiotensin II (Ang II) in mice.

**Methods and Results:**

Cat S-knockout (Cat S^−/−^) and littermate wild-type (WT) C57BL/6J mice were infused continuously with Ang II (750 ng/kg/min) or saline for 7 days. Cat S^−/−^ mice showed severe cardiac fibrosis, including elevated expression of collagen I and α-smooth muscle actin (α-SMA), as compared with WT mice. Moreover, macrophage infiltration and expression of inflammatory cytokines (tumor necrosis factor α, transforming growth factor β and interleukin 1β) were significantly greater in Cat S^−/−^ than WT hearts. These Ang II-induced effects in Cat S^−/−^ mouse hearts was associated with abnormal accumulation of autophagosomes and reduced clearance of damaged mitochondria, which led to increased levels of reactive oxygen species (ROS) and activation of nuclear factor-kappa B (NF-κB) in macrophages.

**Conclusion:**

Cat S in lysosomes is essential for mitophagy processing in macrophages, deficiency in Cat S can increase damaged mitochondria and elevate ROS levels and NF-κB activity in hypertensive mice, so it regulates cardiac inflammation and fibrosis.

## Introduction

Hypertensive heart disease is an inflammatory disease characterized by extensive fibrosis and remodeling of the heart. The fibrotic heart leads to stiffening of the ventricles and impaired diastolic function and causes heart failure [1]. Therefore, understanding the mechanisms of fibrosis development in the heart during hypertension is pivotal to find new therapies to counteract heart failure.

Accumulating evidence suggests that inflammation is a key mechanism contributing to cardiac fibrosis in hypertension and other heart diseases [2]. Myocardial infiltration of pro-inflammatory cells such as activated macrophages is the major event of hypertensive heart failure. These cells release various pro-inflammatory cytokines, including tumor necrosis factor (TNF)-α, interleukin (IL)-1, IL-6 and adhesion molecules [3], [4], which stimulates the differentiation of resident cardiac fibroblasts into myofibroblasts and then produces excessive extracellular matrix components, thus leading to cardiac fibrosis [5]. However, the intracellular signaling responsible for initiating inflammation is unclear.

Recently, inducible cathepsin (Cat), of the cysteine protease family, including S, K, C, V, and W, have been shown to play important roles in inflammatory and/or autoimmune diseases such as cancer [6], rheumatoid arthritis [7], osteoporosis, abdominal aortic aneurysm and atherosclerosis [8], [9]. Cat S expression was upregulated after balloon angioplasty in hypercholesterolemic rabbits [10]. Cat S and K are highly expressed in human atherosclerotic and aneurystic lesions [11]. Moreover, angiotensin II (Ang II) was found to stimulate the mRNA expression of Cat S and macrophage-mediated collagenolytic and elastolytic activities [12] and directly modulated inflammation and apoptosis of smooth muscle cells in atherosclerosis.

Cathepsins localize in lysosomes and endosomes and function outside to degrade unwanted intracellular or endocytosed proteins. Several investigations have found accumulation of abnormal vacuolar structures in brain neurons of Cat D^−/−^ or Cat B^−/−^/Cat L^−/−^ mice [13], [14], [15], [16], which suggests that these proteases can regulate autophagy. Studies have demonstrated that activation of the Ang II/AT1 receptor system stimulates cardiomyocytes and podocytes to activate autophagy [17], [18], and investigations have found cardiac autophagy significantly increased with aortic band-induced pressure overload [19]. However, whether Cats contribute to cardiac inflammation and fibrosis in Ang II-induced hypertension is unknown.

In this study, we aimed to determine whether Cat S protease can regulate extracellular matrix and fibrosis induced by hypertension. Cat S-knockout (Cat S^−/−^) mice showed increased cardiac fibrosis, macrophage infiltration and expression of inflammatory cytokines. The mechanism is associated with accumulation of autophagosome and defects in processing mitophagy in lysosomes, thus leading to increased mitochondrial production of ROS and activation of nuclear factor-κB (NF-κB) inflammatory signaling.

## Materials and Methods

### Ethics Statement

Male Cat S-/- and wild-type (WT) mice in a C57BL/6 background were bred and maintained in the Laboratory of Animal Experiments at Capital Medical University. The mice were given a standard diet. The investigations conformed to the US National Institutes of Health Guide for the Care and Use of Laboratory Animals (publication no. 85–23, 1996) and were approved by the Animal Care and Use Committee of Capital Medical University.

### Mouse Model of Ang II-Induced Hypertension

Mice were anesthetized by intraperitoneal injection of pentobarbital (70 mg/kg). Hypertension was induced in Cat S^−/−^ and WT mice (n = 8 per group) by subcutaneous infusion of Ang II at 750 ng/kg/min for 7 days via an Alzet Mini-osmotic Pump (MODEL 1007D, DURECT, Cupertino, CA) [20]. Control animals received saline infusion only. Systolic blood pressure (SBP) and heart rate before and at day 6 after treatment were measured by tail-cuff sphygmomanometry (BP-98A, Softron, Japan). Mice were anaesthetized with 2% to 4% isofluorane and underwent echocardiography in M-mode performed in triplicate [21] by use of the Vevo 770 High Resolution Imaging System (Visual Sonics Inc. Toronto, Canada).

### Histology and Immunohistochemistry

All animals were euthanized 7 days after Ang II or saline infusion by an overdose of pentobarbital (100 mg/kg), hearts were perfused with heparin (in saline), collected and fixed in 4% paraformaldehyde solution and frozen. Fixed hearts were cut into 5-µm sections and stained with hematoxylin and eosin by standard procedures. To measure fibrotic areas, sectioned hearts were stained with Masson trichrome. The interstitial fibrotic areas were calculated as the ratio of total interstitial fibrosis area to total section area by use of a NIS-ELEMENTS quantitative automatic program (Nikon, Japan). Sections underwent immunohistochemical staining with the antibodies against Cat S (1∶300) and collagen I (1∶800) (both Abcam, Cambridge, MA), α-smooth muscle actin (α-SMA; 1∶200), Mac-2 (1∶400), IL-1β (1∶200), TNF-α (1∶200), and TGF-β (1∶200) (all Santa Cruz Biotechnology, Santa Cruz, CA). Images were captured with use of a Nikon Labophot 2 microscope equipped with a Sony CCD-Iris/RGB color video camera attached to a computerized imaging system and analyzed by use of ImagePro Plus 3.0 (ECLIPSE80i/90i; Nikon, Tokyo, Japan) with blinding to treatment.

### Immunofluorescence Staining and Fluorescence Imaging

Frozen heart sections underwent double immunofluorescence staining with primary antibodies against LC3 (1∶200) (MBL International, Woburn, MA), Cat S (1∶200) and F4/80 (1∶200) (both Abcam), and NF-κB p65 (1∶150) (Santa Cruz Biotechnology) at 4°C overnight and Alexa Fluor 488-conjugated or 594-conjugated secondary antibodies (Molecular Probes, Carlsbad, CA). The slides were mounted in glycerol-based vectashield medium (Vector Laboratories, Burlingame, CA) containing 4-,6-diamidino-2-phenylindole (DAPI) for nuclear staining. RAW 264.7 cells were transfected with Cherry-GFP-LC3 (1 µg) using Lipofectamine 2000 (Invitrogen) for 48 hr [22]. Cat S inhibitor (Cbz-Phe-Leu-COCHO) (Calbiochem, Germany) was used (15 nmol/L) for 12 hr to inhibit endogenous Cat S.

### Western Blot Analysis

Proteins were extracted from macrophages or heart tissues and underwent western blot analysis as described [23]. The membranes were incubated with primary antibodies against LC3 (1∶500) (MBL International) at 4°C overnight and then with IRDye-conjugated secondary antibodies (1∶5000; Rockland Immunochemicals, Gilbertsville, PA) for 1 hr. Images were quantified by use of the Odyssey infrared imaging system (LI-COR Biosciences, Lincoln, NE, USA). Levels of proteins were normalized to that of GAPDH.

### RNA Extraction and Real-time PCR

RNA was extracted from mouse heart tissues by the Trizol method (Invitrogen, Carlsbad, CA), and 2 µg RNA was reversed transcribed with MMLV and oligo (dT) primer (Invitrogen). Real-time quantitative PCR involved use of the Bio-Rad iQ5 system (Bio-Rad) with SYBR Green I (Takara, Shiga, Japan). GAPDH was used as a control. Primers were for TGF-β1, 5′-CAACAATTCCTGGCGTTACCTTGG-3′ and 5′-GAAAGCCCTGTATTCCGTCTCCTT-3′; IL-1β, 5′-CTTCAGGCAGGCAGTATCACTCAT-3′ and 5′-TCTAATGGGAACGTCACACACCAG-3′; TNF-α, 5′-CATGAGCACAGAAAGCATGATCCG-3′ and 5′-AAGCAGGAATGAGAAGAGGCTGAG-3′; and GAPDH, 5′-CCTGGAGAAACCTGCCAAGTATGA-3′ and 5′-TTGAAGTCACAGGAGACAACCTGG-3′.

### Macrophage Extraction, Culture and Treatment

Treated mice were injected with 1.5 ml of 3.85% thiogycollate 3–5 days before macrophage isolation. Peritoneal macrophages were obtained by lavaging the peritoneal cavity with 5 ml of 10 mM phosphate-buffered saline (PBS), cells underwent hemocytometry and plating in 6-well plates at 3×10^6^ cells per well, then culture in DMEM containing 10% fetal bovine serum. After 4 hr, non-adherent cells were removed by changing the medium. Cell preparations contained more than 95% macrophages. For Ang II treatment, cells were washed with PBS and incubated with Ang II (100 nM) for 48 hr.

For transmission electron microscopy (TEM), cells were fixed in 2% glutaraldehyde in 0.1 M sodium cacodylate buffer (pH 7.0) for 2 hr, post-fixed in 2% osmium tetroxide for 2 hr, dehydrated with increasing concentrations of ethanol, and gradually infiltrated with Araldite resin.

### Intracellular ROS Production and Mitochondrial ROS Localization

MitoTracker (Invitrogen) was used to label mitochondria. Isolated macrophages were incubated with MitoTracker for 20 min in a light-protected humidified chamber at 37°C before analysis by confocal fluorescence microscopy (Leica) and ImagePro Plus 3.0 with blinding to treatment. MitoTracker and LC3 co-localization was used to detect the mitochondria autophagy (mitophagy).

MitoSOX (Invitrogen) was used to track mitochondria-specific ROS production. Isolated macrophages were incubated with MitoSOX (5 µM) for 15 min in a light-protected humidified chamber at 37°C and washed with PBS for 3 times before analysis by confocal fluorescence microscopy. Flow cytometry was performed using excitation/emission of 488/625 nm for MitoSOX.

### NF-κB Luciferase Reporter Assay

NF-κB transcriptional activity of macrophages was measured after adenovirus infection with NF-κB luciferase reporter. Wild type (WT) or Cat S^−/−^ macrophages were infected for 24 hr before Ang II stimulation. Macrophages were seeded in 24-well plates at 4×10^5^ per well. In total, 100 µl 2× luciferin was added to wells for 3 min, then NF-κB activity (Total Flux) was analyzed by the Xenogen IVIS Imaging system and LivingImage 2.11 (Carliper Corp., Alameda, CA).

### Statistical Analysis

Data are expressed as mean ± SEM. Differences between groups were analyzed by Student’s *t* test or ANOVA, then Newman-Keuls test by use of GraphPad Prism 5.0. P < 0.05 was considered statistically significant.

## Results

### Ang II Infusion Increases Cat S Expression in the Heart

**Figure 1:**
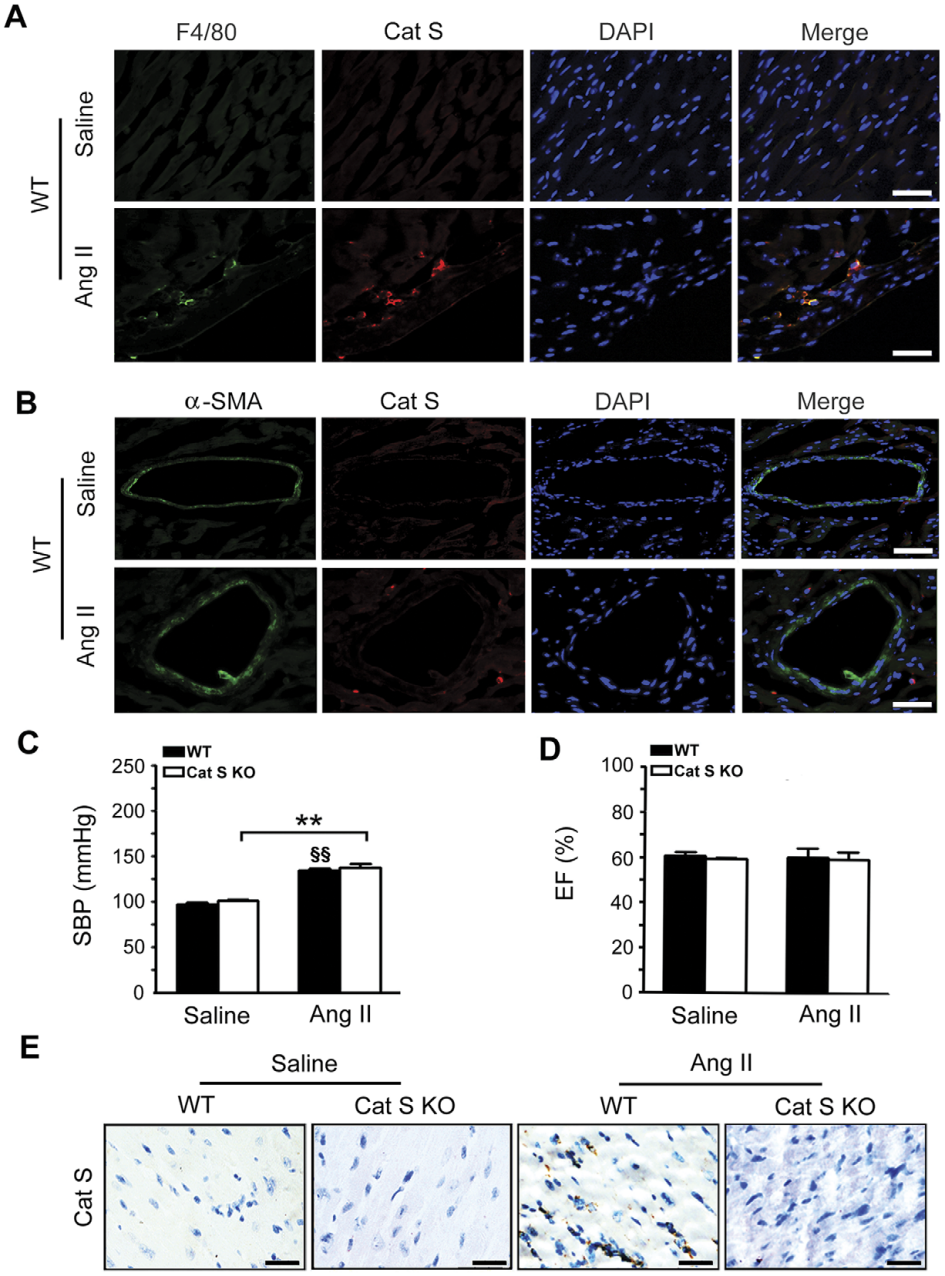
Angiotensin II (Ang II) increases cathepsin S (Cat S) expression in mouse heart. (**A**) Co-localization of Cat S with F4/80-positive macrophages and (**B**) α-smooth muscle actin (α-SMA) in WT and Cat S^−/−^ hearts. Bars, 25 µm. (**C)** Measurement of blood pressure in WT and Cat S^−/−^ mice. (**D**) Left ventricle ejection fraction (EF %) in WT and Cat S^−/−^ hearts. (**E**) Immunohistochemical staining of Ang II-induced Cat S expression in mice. Bars, 25 µm. Data are mean±SEM (n = 8 per group). **P<0.01 *vs*. saline control. ^§§^P<0.01 *vs*. saline control.

To determine the role of Cat S in Ang II-induced cardiac fibrosis *in vivo*, we infused WT and Cat S^−/−^ mice with a low dose, 750 ng/kg/min, of Ang II for 7 days. Double immunofluorescence staining of WT heart sections from mice demonstrated that Ang II infusion induced Cat S co-localization with the macrophage marker F4/80 (Figure 1A) but barely with the SMC marker α-SMA (Figure 1B) in regions of infiltrate inflammatory cells. Therefore, Ang II infusion stimulated the expression of Cat S in cardiac macrophages that may be involved in Ang II-induced cardiac fibrosis.

Baseline systolic blood pressure and left ventricular ejection fraction were similar for both WT and Cat S^−/−^ mice (Figure 1C and D). After Ang II infusion, systolic blood pressure was moderately increased, with no difference between the groups. Immunohistochemistry revealed Cat S expression increased with Ang II (Figure 1A and 1E). As a control, Cat S^−/−^ mice showed no Cat S expression (Figure 1E).

### Ang II-induced Cardiac Fibrosis Enhanced with Cat S Knockout

**Figure 2:**
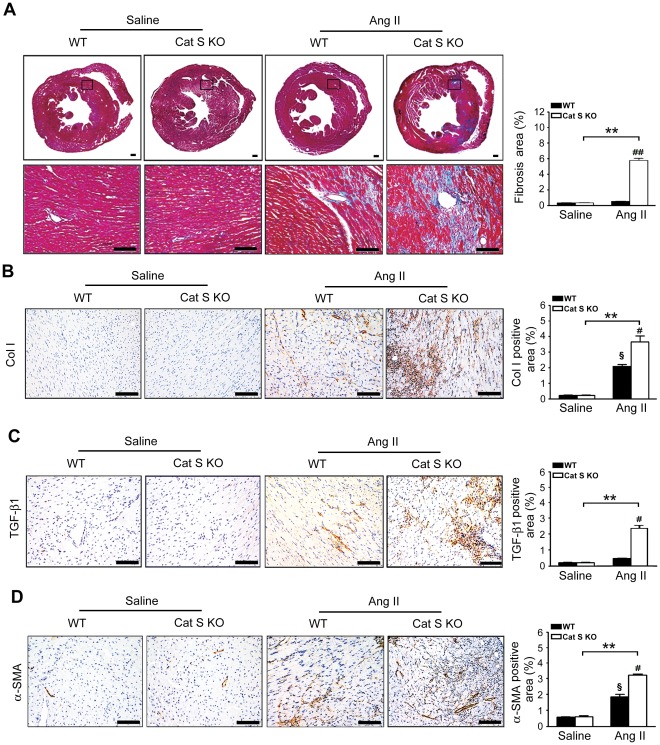
Cat S deficiency enhances Ang II-induced cardiac fibrosis in mouse heart. (**A**) Representative Masson trichrome staining of WT and Cat S^−/−^ hearts with saline or Ang II infusion and quantitative analysis of fibrotic areas. Immunohistochemical staining and quantification of (**B**) collagen I, (**C**) transforming growth factor (TGF)- β1 (**D**) and (**E**) α-SMA in WT and Cat S^−/−^ hearts with saline or Ang II infusion. Bars, 50 µm. Data are mean±SEM (n = 4 per group). **P<0.01 *vs.* saline Cat S KO control; ^#^P<0.05, ^##^P<0.01 *vs.* Ang II-infused WT mice. ^§^P<0.05 *vs.* saline WT control.

To examine whether Cat S deficiency affects Ang II-induced cardiac fibrosis, WT and Cat S^−/−^ mice were infused with Ang II or saline for 7 days and fibrosis was detected by Masson trichrome staining. As compared with saline infusion, Ang II infusion markedly increased the fibrotic areas in WT hearts, which was further increased in Ang II-treated Cat S^−/−^ hearts (Figure 2A). Ang II infusion also significantly increased the expression of collagen I (Figure 2B) and TGF-β1 (Figure 2C) in WT hearts, which was further increased in Cat S^−/−^ hearts. Furthermore, the expression of α-SMA, a myofibroblast marker, was markedly higher in Cat S^−/−^ than WT hearts (Figure 2D). Thus, Cat S plays a critical role in regulating cardiac fibrosis.

### Inflammatory Response Increased with Cat S Knockout

**Figure 3:**
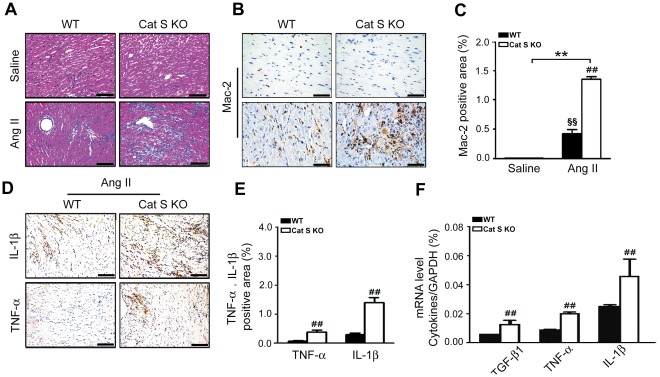
Cat S deficiency increases Ang II-induced infiltration and expression of proinflammatory cytokines in mouse heart. (**A**) Representative hematoxylin and eosin staining of WT and Cat S^−/−^ hearts. Bars, 50 µm. (**B**) Immunohistochemical staining and (**C**) quantification of Mac-2 positivity. Bars, 25 µm. (**D**) Immunohistochemical staining and (**E**) quantification of TNF-α and IL-1β expression. Bars, 50 µm. (**F**) Real-time PCR analysis of mRNA expression of TGF–β1, TNF-α and IL-1β in WT and Cat S KO mouse hearts. Bars, 50 µm. Data are mean±SEM (n = 4 per group). **P<0.01 *vs.* saline Cat S KO control; ^##^P<0.01 *vs.* Ang II-infused WT mice.

Hypertension-induced fibrosis is characterized by left ventricular hypertrophy, cardiac inflammation and collagen deposition, but Cat S has little collagenase activity, our observed cardiac fibrosis in Cat S^−/−^ mice suggests another mechanism for the increased cardiac fibrosis. Inflammatory cell infiltration is an early response of hypertension-induced fibrosis, so we examined whether Cat S deficiency affects the accumulation of proinflammatory cells in hearts, thus leading to fibrosis. Ang II infusion markedly increased the infiltration of inflammatory cells in WT hearts: the number of inflammatory cells (increased nucleus) was higher in Cat S^−/−^ than WT hearts (Figure 3A), and the number of Mac-2-positive cells, a marker of macrophages, was significantly higher in Cat S^−/−^ than WT hearts (Figure 3B). After Ang II infusion, the expression of proinflammatory TNF-α and IL-1β, and TGF-β1, was markedly higher in Cat S^−/−^ than WT hearts (Figure 3D-F).

### Cat S Deficiency Induces ROS Production and NF-κB Activation

**Figure 4:**
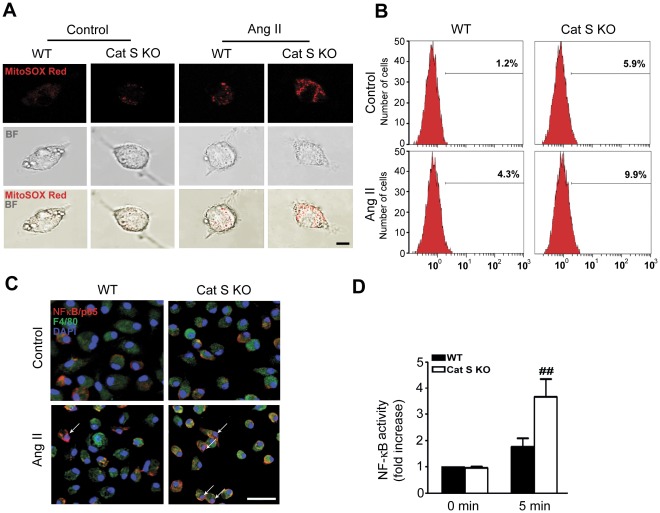
Cat S deficiency increases Ang II-induced reactive oxygen species (ROS) production and NF-κB activation in macrophages. (**A**) Confocal microscopy revealed MitoSOX Red fluorescence of mitochondria-specific ROS production and colocalization with bright field (BF), with and without Ang II treatment for 2 hr. Bars, 2 µm. (B) Quantification of MitoSOX Red positive macrophages (%) by flow cytometry. (C) Immunohistochemical staining of NF-κB/p65. Bars, 10 µm (**D**) Luciferase assay of NF-κB activity. Data are mean±SEM (n = 6 per group). **P<0.05 *vs.* Cat S KO control macrophages.^ ##^ *P*<0.01 *vs.* Ang II-treated WT macrophages. ^§§^ P<0.01 *vs.* WT control macrophages.

Elevated Ang II can stimulate mitochondria to produce large amounts of ROS, thus leading to inflammation in vascular cells [24]. We have used the MitoSOX Red fluorescence assay for mitochondria-specific ROS. As shown in Figure 4A, the level of red fluorescence (ROS production) was significantly increased in mitochondria of Cat S-/- macrophages after Ang II (1 µM) treatment as compared with that of WT macrophages. Moreover, MitoSOX Red-positive cells were also detected by flow cytometry, lack of Cat S increased amount of positive cells (Figure 4B). These results suggested that Cat S has an important role in regulating mitochondrial specific ROS production.

ROS is an important trigger of NF-κB activation, a major transcriptional factor inducing inflammatory gene expression. To link Cat S deficiency with aggravated cardiac inflammation, we detected phosphorylated NF-κB/p65 in mouse macrophages. Phosphorylation of p65 was greater in Cat S^−/−^ than WT macrophages with Ang II stimulation (Figure 4C). Finally, to link phosphorylated NF-κB/p65 to its transcriptional activity, we infected isolated macrophages with a recombinant adenovirus containing NF-κB-Luc reporter. Ang II induced NF-κB luciferase activity in WT macrophages (Figure 4D), which was significantly increased in Cat S^−/−^ macrophages.

### Cat S Deficiency Increases the Accumulation of Autophagosomes in Macrophages

**Figure 5:**
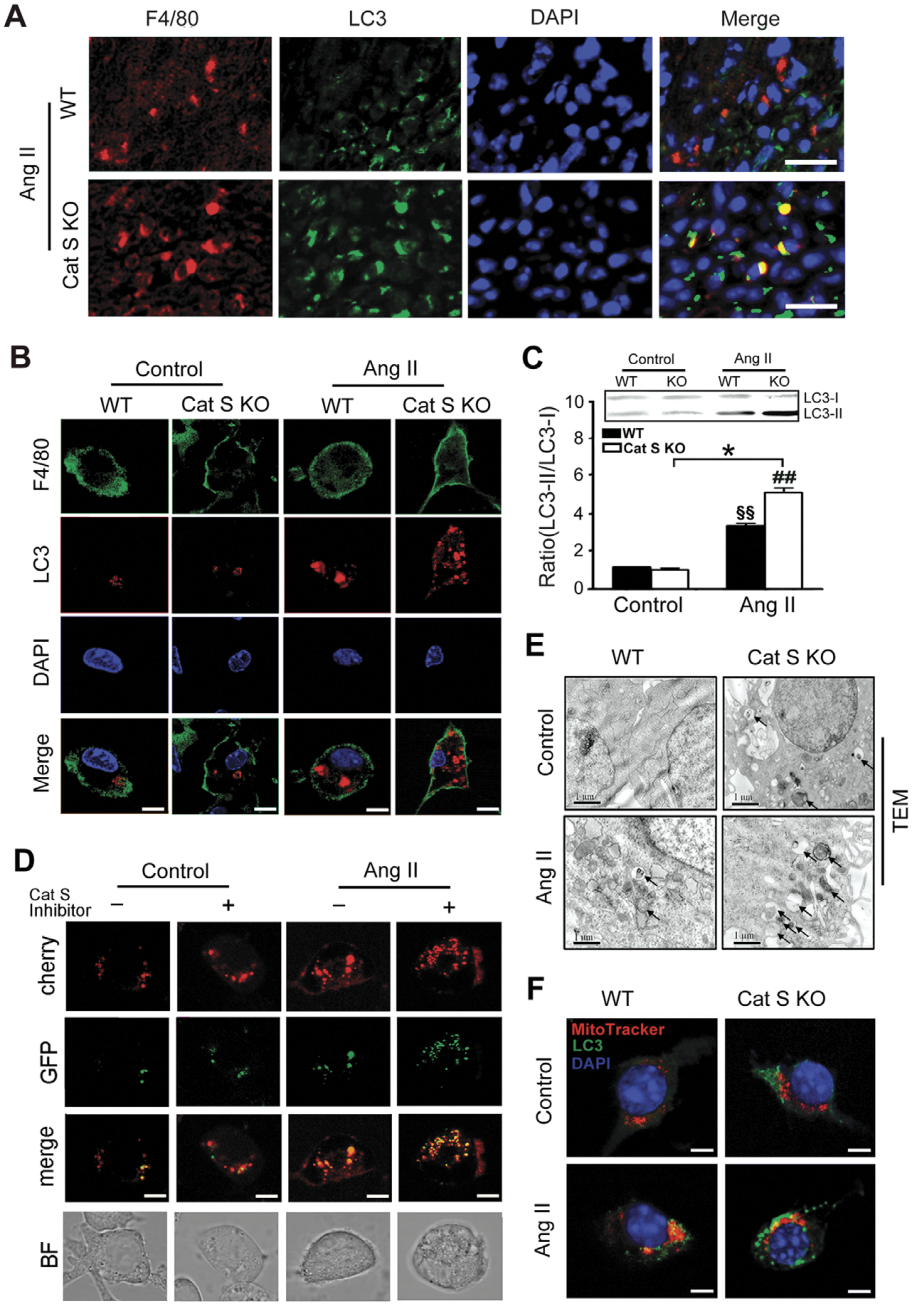
Cat S deficiency increases the Ang II-induced accumulation of autophagosomes in mouse heart. Immunostaining for LC3 and F4/80 in (**A**) mouse heart (Bars, 10 µm) and (**B**) macrophages (Bars, 5 µm). (**C**) Western blot analysis of LC3 protein expression in macrophages. (**D**) RAW 264.7 cells were transfected with Cherry-GFP-LC3, and treated with and without Ang II in the presence or absence of Cat S inhibitor (15 nmol/L). Confocal fluorescence was used to image autophagosomes (red and green foci) and autolysosomes (red-only foci). Bars, 5 µm. (**E**) Representative electron microphotographs of cytoplasmic vacuolization in macrophages treated with saline or Ang II (100 nM) for 48 hr (n = 3 per group). Bars, 1 µm. (**F**) Immunostaining for LC3 and mitochondria marker MitoTtracker in macrophages. Bars, 2 µm. Data are mean±SEM (n = 4 per group). *P<0.05 *vs.* Cat S KO control macrophages.^ ##^ *P*<0.01 vs. Ang II-treated WT macrophages. ^§§^ P<0.01 *vs.* WT control macrophages.

Several studies showed that damaged mitochondria release ROS, which could be suppressed by macro-autophagy of mitochondria (mitophagy) [25]. Activation of the Ang II/AT1 receptor system stimulates cardiomyocytes and podocytes to activate autophagy [17], [18], so we tested whether elevated ROS in the Ang II-treated Cat S^−/−^ heart could be linked to mitophagy. On immunofluorescence staining, Ang II infusion was found to induce co-localization of the autophagosomal marker LC3 (Atg8) [26] and macrophage marker F4/80 in WT hearts, and this effect was significantly increased in Cat S^−/−^ hearts (Figure 5A). To confirm these *in vivo* results *in vitro*, confocal analysis showed that Ang II treatment markedly stimulated LC3 protein clustering in WT macrophages, which was further enhanced in Cat S^−/−^ macrophages (Figure 5B). Cleavage of soluble LC3 (LC3-I) to form LC3-II is associated with extent of autophagosome formation [26]. We therefore measured the cleavage of LC3. Ang II treatment induced LC3-II formation in WT macrophages, which was further increased with Cat S deficiency (Figure 5C). Moreover, we have performed an autophagic flux assay to compare basal autophagic flux and Ang II -inducible autophagic flux in wild type cells in the presence or absence of Cat S inhibitor. We used CMV-cherry-GFP-LC3, which displays dual red-green fluorescence in autophagosomes but loses GFP signal in the acidic environment of autolysosomes [22] for cherry-GFP-LC3 flux. As shown in Figure 5D, the cherry-GFP-LC3 flux assay demonstraed Cat S deficiency increased the autophagosome formation in macrophages.

Cytosolic autophagic vacuoles or organelles resembling autophagosomes can be detected by TEM. TEM revealed cytosolic autophagic vacuole formation in a number of WT macrophages, which was exaggerated in Cat S^−/−^ macrophages (Figure 5E). WT and Cat S^−/−^ macrophages did not differ in formation of autophagosomes under saline conditions (Figure 5B-F). To link Cat S deficiency to abnormal autophagy in mitochondria, we performed immunofluorescence analysis and found MitoTracker colocalized with LC3 (Figure 5F), which indicates that deficiency in Cat S leads to increased accumulation of mitophagy in macrophages.

## Discussion

Cat S is a lysosomal protease expressed in human atherosclerotic and aneurysmal tissues that may contribute to inflammatory diseases. Clearance of damaged mitochondria prevents ROS production and inflammation [27].We aimed to define the role of Cat S in Ang II-induced cardiac inflammation and fibrosis. Ang II infusion increased Cat S expression in macrophages, and Cat S knockout led to significant accumulation of autophagosomes, increased macrophage production of mitochondrial ROS and activation of NF-κB, which led to macrophage infiltration and expression of pro-inflammatory cytokines and, subsequently, cardiac fibrosis.

Cat S has elastolytic and collagenolytic properties [9], [10] and is highly expressed in atherosclerotic lesions and aneurysms. Cat S is expressed mainly in macrophages, smooth muscle cells, endothelial cells and internal elastic lamin fragmentation but not in healthy human aortas [28]. Incubation of these cells with inflammatory cytokines such as TNF-α, IL-1β and interferon γ (IFN-γ) significantly induced the expression and secretion of Cat S [28]. Our results revealed that Ang II treatment markedly increased Cat S expression in macrophages but little in smooth muscle cells in heart tissue (Figure 1).

Hypertensive heart disease is characterized by left ventricular hypertrophy, cardiac inflammation and fibrosis. Inflammation plays an important role in the initiation and development of cardiac remodeling. Infiltration and activation of macrophages is a key step in the pathogenesis of cardiac fibrosis [29]. However, the signaling mechanisms that activate macrophages are not fully understood. Increasing evidence suggests that Cathepsins may have a direct or indirect role in regulating the expression of proinflammatory cytokines. For example, Cat K deficiency blocked inflammation by enhancing the expression of profibrotic factors such as TGF-β in atherosclerotic lesions of Apo E^−/−^ mice [30]. Cat S deficiency attenuated the infiltration of macrophages and T cells, IFN-γ expression and lipid content in Apo E^−/−^ lesions, the elastase activity of Cat S caused the damage in atherosclerosis [31].

Several studies have revealed an important role for autophagy in the cardiomyocyte reaction to numerous types of stress, and compelling evidence has emerged that this autophagic response participates in the pathogenesis of disease [32]. For example, Kostin et al. examined explanted hearts from patients with end-stage heart failure and found evidence for each of the three major types of cell death, with autophagic death as the prominent one [33]. Knaapen et al. reported that as many as 0.3% of cardiomyocytes in end-stage failing human hearts display features of autophagy [34]. Similar to our study, In pressure overload heart failure, autophagic activity is rapidly induced [19]. Nakai et al reported that basal autophagy is required for normal cardiac function and that upregulation of autophagy is an adaptive response to hemodynamic stress [35]. Investigations have found accumulation of abnormal vacuolar structures in brain neurons of Cat D^−/−^ or Cat B^−/−^/Cat L^−/−^ mice [13], [14], [15], [16]; deficiency of lysosomal enzymes including Cat D, Cat B and Cat L can induce the accumulation of abnormal vacuolar structures in brain neurons, which suggests that these proteases can regulate autophagy. Autophagy is a vacuolar lysosomal degradation pathway for long-lived proteins and damaged organelles that are critical for maintaining cell function under the stress conditions [36], [37]. At the late stage of autophagy, autophagysomes are presented to lysosomes to degrade damaged organelles (i.e., mitochondria) [38]. Autophagy is induced in various cardiovascular diseases, including hypertrophy, myocardial injury and atherosclerosis [32], [39], [40], [41], [42], However, the relationship between lysosomal Cat S and macroautophagy in pathophysiological conditions remain unclear. We found that Ang II infusion significantly induced autophagosome/vacuolar accumulation, characterized by increased LC-3 expression in macrophages of WT hearts; Cat S deficiency enhanced this effect (Figure 5). Thus, Cat S can regulates the accumulation of autophagosomes/mitolysosomes in macrophages with Ang II treatment.

Our results showed that abnormal accumulation of autophagysomes may be responsible for the Cat S deficiency-enhanced inflammation and fibrotic response in the heart. The link could be mitochondria ROS production. Damaged mitochondria releases ROS, which causes the inflammatory response [43]. Numerous studies have demonstrated ROS as important triggers activating NF-κB transcriptional activity and that antioxidants can inhibit NF-κB activation [44], [45]. NF-κB signaling plays a pivotal role in regulating the inflammatory, immune, and apoptotic responses in mammals [46]. We found that Ang II-induced ROS production and phosphorylation of NF-κB/p65 and NF-κB activity was greater in Cat S^−/−^ than WT mouse macrophages (Figure 4). Defective Cat S triggered abnormal mitophagy (mitochondria colocalized with LC3) (Figure 5), which implies a role of Cat S in regulating ROS production and inflammation. Increasing evidence has demonstrated that Ang II, via its type 1 receptor, activates a number of signaling pathways, including ROS, and transcriptional factors such as NF-κB, Ets-1, and early growth response 1 to regulate cardiac hypertrophy, inflammation, and fibrosis [47]. We found that Cat S deficiency markedly induced macrophage infiltration and the expression of the fibrotic factor TGF-β1 and inflammatory factors TNF-α and IL-1β in mouse cardiac tissue (Figure 3) in response to Ang II treatment, which increased myofibroblasts formation, collagen I deposition and fibrosis (Figure 2). Thus, Cat S is a key factor in initiating inflammation and cardiac fibrosis. However, Zheng et al have reported that selective Cat S inhibition and a null mutation of Cat S decreased IFN-γ-induced DNA injury, apoptosis, emphysema, inflammation, and protease accumulation in epithelial cells. They demonstrated that Cat S-dependent apoptosis is a critical event in the pathogenesis of IFN-γ-induced alveolar remodeling and emphysema [48]. In combine with our results, the role of Cat S may be resulted from the different function of Cathepsins or dual nature of autophagy regarding the difference in stimuli and cells type, which is a recurring theme in other organ systems and disease state [49].

In conclusion, we provide novel insights into the roles of Cat S in regulating cardiac fibrosis and the inflammatory response to Ang II in mouse by modulating mitophagy degradation, ROS production and NF-κB activation. Activation of the lysosomal protease Cat S may be a critical switch for autophagic inflammation and cardiac fibrosis in hypertension.
